# Laboratory Investigations of African Pouched Rats (*Cricetomys gambianus*) as a Potential Reservoir Host Species for Monkeypox Virus

**DOI:** 10.1371/journal.pntd.0004013

**Published:** 2015-10-30

**Authors:** Christina L. Hutson, Yoshinori J. Nakazawa, Joshua Self, Victoria A. Olson, Russell L. Regnery, Zachary Braden, Sonja Weiss, Jean Malekani, Eddie Jackson, Mallory Tate, Kevin L. Karem, Tonie E. Rocke, Jorge E. Osorio, Inger K. Damon, Darin S. Carroll

**Affiliations:** 1 Poxvirus and Rabies Branch, Centers for Disease Control and Prevention, Atlanta, Georgia, United States of America; 2 Department of Biology, University of Kinshasa, Kinshasa, Democratic Republic of Congo; 3 Animal Resources Branch, Centers for Disease Control and Prevention, Atlanta, Georgia, United States of America; 4 U.S. Geological Survey-National Wildlife Health Center, Madison, Wisconsin, United States of America; 5 Department of Pathobiological Science, School of Veterinary Medicine, University of Wisconsin, Madison, Wisconsin, United States of America; The George Washington University School of Medicine and Health Sciences, UNITED STATES

## Abstract

Monkeypox is a zoonotic disease endemic to central and western Africa, where it is a major public health concern. Although *Monkeypox virus* (MPXV) and monkeypox disease in humans have been well characterized, little is known about its natural history, or its maintenance in animal populations of sylvatic reservoir(s). In 2003, several species of rodents imported from Ghana were involved in a monkeypox outbreak in the United States with individuals of three African rodent genera (*Cricetomys*, *Graphiurus*, *Funisciurus*) shown to be infected with MPXV. Here, we examine the course of MPXV infection in *Cricetomys gambianus* (pouched Gambian rats) and this rodent species’ competence as a host for the virus. We obtained ten Gambian rats from an introduced colony in Grassy Key, Florida and infected eight of these via scarification with a challenge dose of 4X10^4^ plaque forming units (pfu) from either of the two primary clades of MPXV: Congo Basin (C-MPXV: n = 4) or West African (W-MPXV: n = 4); an additional 2 animals served as PBS controls. Viral shedding and the effect of infection on activity and physiological aspects of the animals were measured. MPXV challenged animals had significantly higher core body temperatures, reduced activity and increased weight loss than PBS controls. Viable virus was found in samples taken from animals in both experimental groups (C-MPXV and W-MPXV) between 3 and 27 days post infection (p.i.) (up to 1X10^8^ pfu/ml), with viral DNA found until day 56 p.i. The results from this work show that *Cricetomys gambianus* (and by inference, probably the closely related species, *Cricetomys emini*) can be infected with MPXV and shed viable virus particles; thus suggesting that these animals may be involved in the maintenance of MPXV in wildlife mammalian populations. More research is needed to elucidate the epidemiology of MPXV and the role of Gambian rats and other species.

## Author Summary

Post smallpox eradication, *Monkeypox virus* (MPXV) has emerged as the most important human health threat within the *Orthopoxvirus* genus. Sporadic outbreaks of monkeypox within Africa, concern over the potential of the virus to move outside of its natural range, as well as the increasing proportion of unvaccinated people now susceptible to MPXV (due to cessation of smallpox vaccination), makes it important to understand how the virus is transmitted to humans within Africa. Thus far the natural reservoir(s) of MXPV has eluded identification; however several rodent species including African pouched rats (*Cricetomys* spp.) have been implicated as possible reservoirs. *Cricetomys* are often found living in close proximity to humans (and invading homes) and additionally serve as a food source within Africa. Therefore, it is important to utilize laboratory methods to examine the course of MPXV infection in *Cricetomys* and thus determine this rodent species’ competence as a host for the virus. We challenged eight animals with MPXV (4X10^4^ pfu) and assessed clinical symptoms and molecular markers of disease. Our results show *Cricetomys* can be infected with MPXV and shed high loads of virus via multiple routes, supporting the hypothesis that they may be involved in the maintenance and transmission of the virus within Africa.

## Introduction

Currently, 10 species are known in the genus *Orthopoxvirus* (OPXV); 6 of them (*Ectromelia*, *Cowpox*, *Volepox*, *Taterapox*, *Monkeypox* and *Vaccinia virus*) have been shown to circulate in rodent species [1–5]. With the eradication of *Variola virus* (the causative agent of smallpox), *Monkeypox virus* (MPXV) is the OPXV that is most problematic with respect to global public health concerns. Through genotyping techniques, prior studies have identified 2 distinct MPXV clades, termed West African and Congo Basin due to their geographic location [6,7]. Congo Basin MPXV has been shown to be more virulent within both animal models as well as humans [8–12]. Currently, the reservoir host(s) and the ecological parameters surrounding transmission of this zoonotic disease from native African species into local human populations is/are uncertain.

Historically, MPXV was thought to have a relatively narrow range of permissive animal hosts, but subsequent outbreaks in zoological gardens and captive primate colonies expanded our knowledge of animal species that suffer acute MPXV infections [13–15]. Since the early 1970’s, field researchers have conducted ecological investigations that involved the collection of vertebrate animals living in proximity to humans suffering from monkeypox disease. During these investigations, a variety of assays were used to identify several species with serological evidence (anti-OPXV antibodies) of past OPXV infection, including a wide variety of mammalian taxa such as ungulates (hoofed animals), non-human primates, and rodents (including squirrels of various genera, *Cricetomys*, *Graphiurus*, and shrews to name a few) [16–20]. However, little success was achieved in determining which of these species or groups of species was responsible for maintaining MPXV in its native range.

In the Democratic Republic of Congo (former Zaïre) the first wild caught animal actively infected with MPXV was captured in1985, a squirrel identified in the field as a Thomas’s rope squirrel (*Funisciurus anerythrus*) [2]. This discovery led to focused research targeting rodents as the potential “reservoir host” species of MPXV in Africa and the conclusion that squirrels were the likely reservoir and that terrestrial rodents may not play a great role in the maintenance or circulation of MPXV in Africa [19,21]. More recently, MPXV was isolated from a sooty mangabey (*Cercocebus atys*) that was found dead during a long-term monitoring program in Taï National Park, Cote d’Ivoire, which represents the second MPXV isolate obtained from a wild animal [22].

In 2003, several species of imported African rodents from Ghana were involved in the introduction of MPXV into the United States and its spread into captive North American prairie dogs (*Cynomys ludovicianus*), and subsequently to humans. Specifically, viable virus was found in the tissues of individuals of three African rodent genera (*Cricetomys*, *Graphiurus*, *Funisciurus*) and additionally, *Graphiurus* showed evidence of maintaining a persistent infection [23]. Unfortunately, it could not be determined at what point during the outbreak these three genera became infected; therefore it is not possible to confidently determine if any of these genera served as the index case species, only that they are susceptible to MPXV infection. Subsequent field research in Ghana Africa of wild-caught rodents revealed anti-OPXV antibodies in four genera and evidence of OPXV DNA in three genera. In only two of the genera studied (*Cricetomys* and *Graphiurus*) was it possible to detect anti-OPXV antibodies and OPXV DNA [20].

Members of the genus *Cricetomys* are native to the savannahs and rain forests of tropical Africa, they dig burrows for shelter and food storage; and can reach body lengths of >67cm and weights of >730 grams [24]. These species are commonly exploited as bushmeat [25,26]. A recent taxonomic revision of the genus divided the previously recognized species and identified three new species; with this, the distribution of *C*. *gambianus* is limited to the savannah region south of the Sahel from the coast of Gambia and Senegal east through Guinea, Côte d’Ivoire, Ghana, Togo, Benin, Nigeria Cameroon and Central African Republic [27]. *C*. *gambianus* were introduced to the Grassy Key, Florida due to activities related to the exotic pet trade [28].

Thus far, virtually every short-term ecological study targeting animals in MPXV endemic areas and elsewhere have had a high degree of success in collecting mammals with evidence of OPXV infection, but the potential cross reactivity of OPXV assays and the difficulty in obtaining wild-caught viremic animals has confounded searches for the true reservoir(s) [16,17,29]. However, based on data from the US and African outbreaks of MPXV, *Cricetomys* has emerged as a rodent genus of interest in the search for the sylvatic source of the disease [16,20,23].

In the current study, we conducted a laboratory challenge study to examine the effects of MPXV infection in *Cricetomys gambianus* to assess its capability to maintain prolonged infections with MPXV and to shed infectious virus at levels that could lead to transmission within and between other rodent species or humans. Additionally, we compared the effects of the infection with West African (W-MPX) and Congo Basin (C-MPX) clades of MPXV in this native African rodent in terms of difference in disease presentation (if any) as is the case with human monkeypox disease.

This work complements previous studies which have similarly examined African squirrels and dormice [30,31] as well as studies of non-African species such as prairie dog, ground squirrels and inbred mice [11,32–38] as potential models for human monkeypox disease. The investigations, including the one described herein, involving African rodents represent laboratory based ecological investigations which complement the early and current field efforts of several research groups meant to elucidate elements surrounding the maintenance and ecology of MPXV in its endemic (African) range.

## Materials and Methods

### Ethics Statement

Permission was obtained from the Food and Drug Administration (FDA) to capture, transport and use these animals in an experimental study at the Centers for Disease Control and Prevention (CDC). All animal handling followed an existing CDC Institutional Animal Care and Use Committee (IACUC)-approved protocol (1376REGRATC). Animals were fully anesthetized in their cage using 5% inhalant isoflourane prior to any manipulation or sampling procedures.

### Study Animals

We followed standard procedures regarding sampling animals potentially infected with viral zoonoses in field collections [39]. All animal handling followed an existing Centers for Disease Control and Prevention (CDC) Institutional Animal Care and Use Committee (IACUC)-approved protocol (1376REGRATC). *Cricetomys* used in this study were caught in Grassy Key, Florida; where a population of this species became established after its introduction to the area via the exotic pet industry (originally purchased from West African populations) described in more detail elsewhere [28]. Serum was collected from the study animals to confirm the absence of anti-OPXV antibodies via ELISA) Described below in Serology.

### Biotelemetry

Two weeks prior to the start of the study, animals were completely anesthetized with 5% inhalant isoflourane and implanted with vital-view mini-mitter IP biotelemetry G2 transmitters following the manufacturer’s guidelines. The telemetry systems were set up to record the activity level of each rat as the number of position changes, and core body temperature measurements beginning one week prior to inoculation and continued at 30 minute intervals throughout the study. For analysis, the temperature and activity for each animal on each sampling day was calculated by averaging all measures (collected every 30 minutes) for each variable so that one average value per sampling day per animal could be compared. Additionally, averages and standard deviations for these variables were calculated for the different groups (experimental and control groups) per sampling day.

### Study Design

Animals were cared for in accordance with CDC IACUC guidelines under an approved protocol (1376REGRATC). Ten *Cricetomys* were divided into two experimental groups of four animals each (C-MPX and W-MPX) and one PBS control group with two animal. Animals were individually housed in cages with wire tops and aerosol barrier lids and received fresh food and water daily. The cages were placed on a metal shelving unit inside a (negative pressure) “holding” Bioclean unit.

Animals were inoculated by a sub-dermal “scarification” route with the appropriate virus (C-MPX or W-MPX) by placing 10l of viral preparation on shaved skin between the scapulae, and lightly pricking the skin 10 times with a 28 gauge needle. The control animals were sham infected using 10l of sterile PBS and housed in adjacent cages on 2 shelves within a Bioclean unit inside of a BSL-3 animal suite. The West African and Congo Basin MPXV strains used in this study were isolated during the 2003 U.S. outbreak (MPXV-2003-044; West African) and an outbreak in the Republic of the Congo in 2003 (MPXV-2003-358; Congo Basin) [7,40]. Both viruses had undergone two passages in African green monkey kidney cells (BSC-40; originally purchased from ATCC and currently maintained by CDC Biologics Information and Ordering System) prior to seed pool production. Sucrose-cushion purified preparations [41] of virus were used for animal challenges. Inocula titers were immediately re-confirmed by standard plaque assay and found to be 4X10^4^ pfu (in a total volume of 10l) for both MPXV clades.

On sampling days, sealed cages were transferred to a clean “processing” Bioclean unit which was closed (under negative pressure) and animals were fully anesthetized in their cage using 5% inhalant isoflourane prior to their manipulation. Once the animal was unconscious, it was weighed and placed on a stainless steel heating surface to maintain normal body temperature and the face was placed in a “nose-cone” to maintain complete anesthesia during sampling. Blood, oral, nasal and rectal swabs were collected on days 0, and every third day through day 21 p.i. (8 samples). After day 21 p.i. sampling intervals were increased to one sample day per week. All animals were euthanized on day 70 p.i., except for one experimental animal that died on day 13 p.i.. Tissue samples were collected from all animals including the following: liver, lung, heart, kidney, spleen, skin, primary lesion and mesenteric lymph node.

These challenge experiments were conducted in a Biological Safety Level 3+ laboratory at the CDC in Atlanta, Georgia. All individual cages were housed in a negative pressure Bioclean unit within a BSL-3 animal suite.

### Sample Preparation

DNA from blood samples was extracted using the EZ-1 DNA extraction robot (Qiagen) from 200l of blood after one hour incubation at 55°C to inactivate virus. To recover DNA from swabs (oral, nasal, rectal, scarification site lesion), 400l of PBS was added to each swab and the swab extraction tube systems (SETS; Roche) protocol was followed to recover a homogenate. DNA from swab samples was obtained from 100l of the swab homogenate (after the homogenate was incubated with Proteinase K and Buffer G2 at 55°C to inactivate virus) using EZ-1 DNA extraction robot (Qiagen); the remaining swab homogenate was used for virus isolation (see below). Tissue samples were placed in disposable dounce homogenizers with 1 ml of PBS and ground thoroughly to create a slurry. Genomic DNA was obtained form 100l of tissue slurry (after the slurry was incubated with Proteinase K and Buffer G2 at 55°C to inactivate virus) with EZ-1 DNA extraction robot (Qiagen) and the remaining slurries were used for virus isolation (see below). All sample processing and testing was performed under BSL-2 conditions with BSL-3 work practices.

### Polymerase Chain Reaction

DNA samples prepared from sampled tissues, blood and swabs were tested for the presence of OPXV DNA by PCR using forward and reverse primers and probe designed to be complimentary to regions of the E9L (DNA polymerase) gene that are able to detect all Eurasian OPXVs, except for variola [42]. MPXV DNA (50fg–5pg) was used as a standard curve to allow for DNA quantification, and six wells with water were used as negative controls. Reactions were placed in either an ABI 7900 or ABI ViiA7 real-time PCR system and subjected to the following thermal cycle parameters: 95°C for 10 minutes; then 95°C for 15 seconds and 63°C for 1 minute for 45 cycles.

Representative samples that tested positive in the generic E9L OPX assay were used for an additional clade specific MPXV- real-time PCR assay to add specificity to the OPXV diagnosis. Forward and reverse primers, and probe for this assay were specific to the West African MPXV clade (GSR_WA) or to the Congo Basin MXPV clade (C3L_assay); PCR reactions were as described within Li et al. [43] and were run on an ABI 7500 PCR platform.

### Viable Virus Loads

Specimens testing positive for OPXV DNA by PCR were evaluated for viable virus by tissue culture propagation. Each swab or tissue sample was titrated using 10 fold dilutions of swab eluent or tissue slurry on BSC-40 cell monolayers, incubated at 36^o^ C and 6% CO_2_ for 72 hours, and subsequently stained with crystal violet and formalin to reveal plaques.

### Serology

We used a modified ELISA assay to screen animal samples for presence of anti-OPXV immunoglobulin types A and G as described in Hutson *et al*. [11]. One half of each Microtitreplate (Immulon II; Dynatech) was coated with 0.01 g crude *Vaccinia virus* (Dryvax grown in BSC-40 cells) in carbonate buffer; the other half was coated with an equal volume of BSC-40 cell lysate diluted in carbonate buffer. After an overnight incubation at 4°C, 10 % formalin was applied for 10 min for inactivation. Plates were blocked for 30 min at room temperature with assay diluent [PBS, 0.01 M, pH 7.4 (Gibco)+0.05% Tween-20, 5% dried skim milk, 2% normal goat serum and 2% BSA] followed by three PBST (0.05% Tween-20) washes. Pouched rat sera were diluted in assay diluent (1:100) and added to both sides of the plates. After a 1hr incubation (37°C) they were washed and then a 1:30000 dilution of ImmunoPure A/G conjugate (Pierce) was added to the plates. After incubation (1h at 37°C) and wash, peroxidase substrate was added followed by addition of stop solution (5–15 min later) (Kirkegaard & Perry Laboratories). Absorbance was read on a spectrophotometer at 450nm. Both positive and negative human anti-vaccinia sera were used as assay controls. The mean value of all the BSC-40 cell lysate portion of each plate was used to generate a cut-off value (COV) by two standard deviations. Specimens with values above the COV were considered positive.

### Statistical Analyses

Change of animal weight was calculated as the percent weight difference at each sample day using day 3 p.i. as the base value. We compared core body temperature, weight change and activity between groups (C-MPX vs. Control, W-MPX vs. Control and C-MPX vs. W-MPX) via the Wilcoxon rank-sum test for each sampling day. Additionally, group means of core body temperature, weight loss and activity level were calculated per sample day and a paired comparison between groups was performed using the Wilcoxon signed-rank test to assess differences in these variables throughout the study. Maximum levels of viable virus and average anti-OPXV antibody were obtained from samples collected from animals of the same group and compared using the Wilcoxon rank-sum test to evaluate differences in viable virus shedding between MPXV strains and type of sample (oral, nasal, rectal, primary lesion and secondary lesion swabs). All statistical analyses were performed using the MASS package [44] in R 2.15.1 [45].

### Accession Numbers

The West African and Congo Basin MPXV strains used in this study were isolated during the 2003 U.S. outbreak (MPXV-2003-044; West African) and an outbreak in the Republic of the Congo in 2003 (MPXV-2003-358; Congo Basin), respectively; and have been fully sequenced in previous works (Accession numbers: DQ011157 and DQ011154) [7,40].

## Results

See Table 1.

**Table 1:** Summary of clinical symptoms, day of primary and generalized lesions onset, and day of first detection of immune response in each experimental animal.

	C-MPX 1	C-MPX 2	C-MPX 3	C-MPX 4	W-MPX 1	W-MPX 2	W-MPX 3	W-MPX 4†
**Scarification site lesion (day of onset)**	Day 3	Day 6	Day 6	Day 6	Day 6	Day 6	Day 6	Day 6
**Generalized cutaneous lesions (day of onset/resolved)**	Day 3 / 27	Day 12 / 24	Day 9 / 27	Day 12 / 35	Day 9 / 27	Day 9 / 21	Day 9 / 27	Day 9 / -*
**Clinical symptoms**	Lethargy, weight loss, possible ocular infection, hypopigmentation	, Weight loss, tongue lesions, hypopigmentation	, Weight loss, lesions near both eyes, tongue lesions, hypopigmentation	Hypopigmentation	, Tongue lesions, small lesion on lip, hypopigmentation	Hypopigmentation	Tongue lesions, hypopigmentation,	Weight loss
**Day of anti-OPXV antibody detection**	Day 6	Day 12	Day 12	Day 12	Day 12	Day 12	Day 12	Day 12

* W-MPX 4 died on day 13 p.i., thus, no information is available for this animal after this day.

### Observed Illness

**Fig 1:**
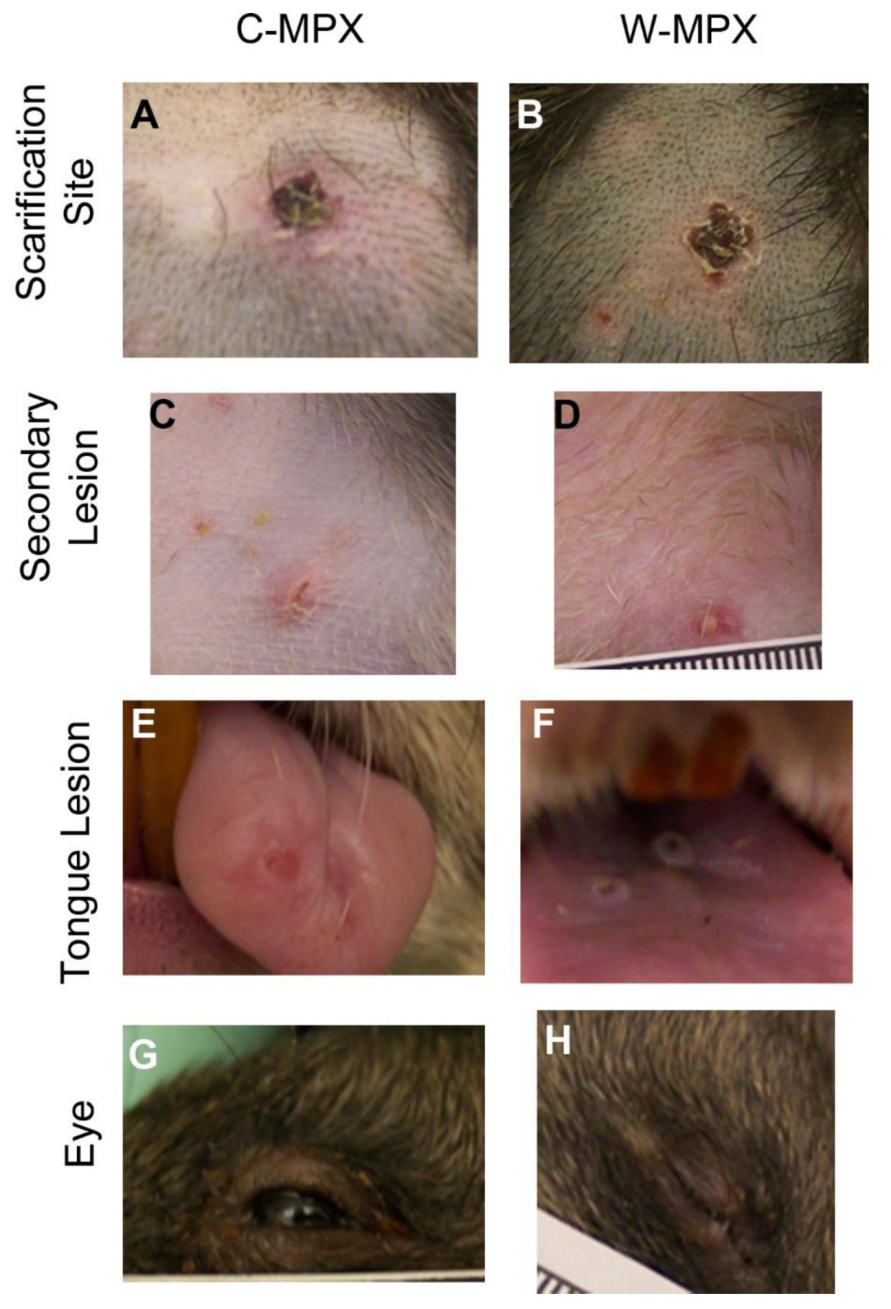
Representative images of cutaneous lesions after experimental challenge with Congo Basin (A, C, E and G) or West African (B, D, F and H) clades of MPXV. (A and B) Pictures of scarification site lesions, (C and D) secondary lesions C and D, (E and F) tongue lesions; and (G and H) eyelid lesions for animals in each experimental group.

All animals in both experimental groups developed obvious cutaneous rash illness with more severe primary lesions at the inoculation site, and less severe disseminated secondary lesions on the dorsal and ventral surfaces of the trunk as well as on the fore and hind limbs (Fig 1). The earliest onset for primary and secondary lesions was day 3 p.i. for one rat in group C-MPX; lesions for this animal began to resolve by day 9 and were fully resolved (only hypopigmented scarring left) by day 27 p.i. All other animals in both experimental groups had distinct primary lesions at the site of scarification by day 6 and distinguishable secondary lesions between days 9–12 p.i. Lesions began to resolve by day 15 and were fully resolved for most animals by day 27, with the exception of one C-MPX challenged animal in which lesions did not completely resolve until day 35 p.i. Two animals in each experimental group developed tongue lesions on day 12 (C-MPX 2 and 3, W-MPX 1 and 3), that resolved by day 18 (C-MPX animals) and 21 p.i. (W-MPX) respectively. Although the gross number of lesions was not noticeably different between viral strains, based on our observations the severity of lesion presentation (size/appearance) seemed more pronounced in animals from group W-MPX than animals from group C-MPX (although this was not a quantifiable measurement). Additionally, animal W-MPX 4 died on day 13 although the overt illness in this animal was not noticeably different from the other animals in its experimental group; tissues samples from this animal were collected during necropsy and tested for OPXV DNA and viable virus. Viral loads in harvested tissues ranged from 3.1X10^6^ (liver) to 2.4X10^9^ (mesenteric lymph node) (Table 2).

**Table 2:** PCR results and viral load for sampled tissues obtained from animal W-MPXV 4 during necropsy.

Sample	PCR (fg/gram)	Viral Load (pfu/gram)
Liver	3.5X10^5^	3.1X10^6^
Lung	4.8X10^5^	4.4X10^6^
Heart	3.0X10^6^	1.2X10^7^
Kidney	4.3X10^5^	1.4X10^7^
Spleen	1.0X10^6^	8.0X10^7^
Skin	6.0X10^5^	9.9X10^7^
Prim.Lesion	4.0X10^6^	2.6X10^8^
Mes.L.N	2.7X10^7^	2.4X10^9^

### Biotelemetry

**Fig 2:**
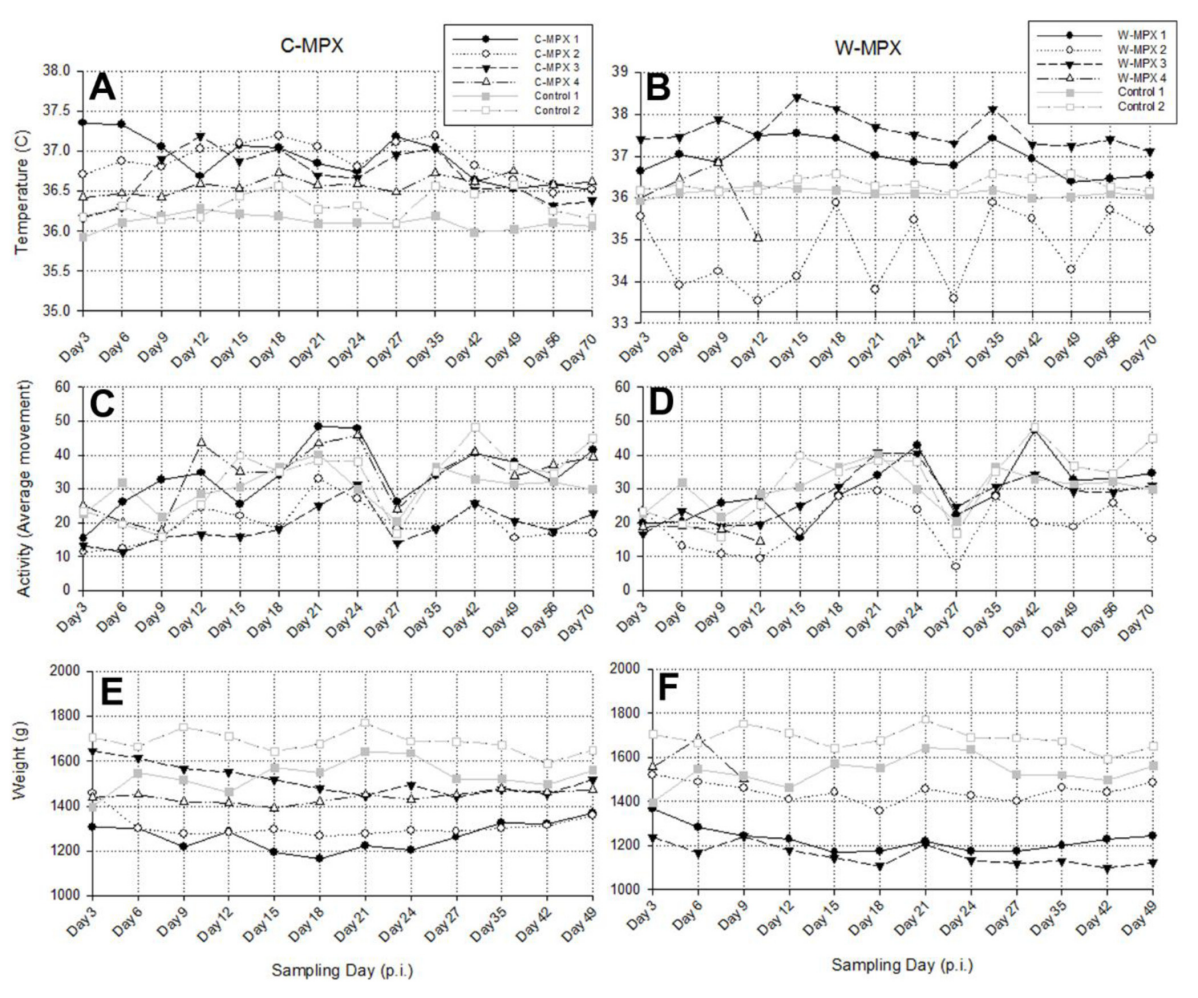
Biotelemetry measurements of temperature (A and B) and activity (C and D); and weight (E and F) of Cricetomys challenged with West African or Congo Basin MPXV. **(A and B)** Recorded core body temperature for experimental and control groups with higher temperatures for animals in C-MPX compared to controls. (C and D) Activity levels showing reduced activity for most experimental animals until day 9 p.i. (C-MPX) and day 12 p.i. (W-MPX), and becoming more active afterwards. (E and F) Weight loss was observed in experimental animals but not in the control group. W-MPX 4 died on day 13 p.i., thus, no information is available for this animal after this day.

There was no significant difference between core body temperatures, activity levels or weight change of experimental animals compared to control animals when compared on a day by day basis (S1 Table). Three animals of group C-MPX and two in group W-MPX showed an increase in temperature following viral challenge (Fig 2). Additionally, when using Wilcoxon signed-rank tests to compare daily average temperatures per group throughout the study, the C-MPX group was significantly higher (p<0.001) than control groups, and significantly higher than the W-MPX group (p = 0.003). The W-MPX average temperatures were marginally significantly higher compared to the control group (p = 0.04).

Animals C-MPX 1 and W-MPX 1 increased their activity after being challenged with MPXV; animals C-MPX 4 and W-MPX 3 showed reduced activity immediately after viral challenge but their activity increased after days 9 and 12 p.i., respectively; the rest of the experimental animals were less active than the control animals throughout the entire study (Fig 2). Comparisons of average activity levels per day throughout the study using Wilcoxon signed-rank test were highly significant between each experimental group and the control group with reduced averages throughout the study for both clades (C-MPX: p = 0.004 and W-MPX: p = 0.001), but no significant difference was found between C-MPX and W-MPX (p = 0.593).

Individual weights and activity were measured throughout the study. All experimental animals lost weight after MPXV challenge and started recovering weight between days 21 and 27 p.i. (Fig 2). Weight loss was statistically higher for both experimental groups compared to the control group (p<0.001 for both groups) and the difference between groups was marginally significant (p = 0.041) with greater weight loss in the C-MPX challenged group.

### Live Viral Particles and Viral DNA (Table 3)

For most animals, the scarification site swab as well as the nasal swab, were the first samples positive for viral DNA at day 3 p.i. Interestingly for one animal within the C-MPX group, all samples were uniformly positive for viral DNA at day 3. For all other animals, oral, rectal and secondary lesion swabs were first positive for viral DNA ranging from day 6–18. Blood samples were only positive for viral DNA for some animals (C-MPX 1, 2 and W-MPX 1, 3, 4); for these animals detection of virus within the blood only lasted for 1–6 days. Not all blood samples were titrated for virus due to lack of adequate sample, therefore we cannot compare the data from titration of a portion of blood samples. Detection of viral DNA was still possible in some animals from a subset of samples (oral, scarification site, nasal swabs) out to day 56 p.i. in both experimental groups (Table 3).

**Table 3:** Range of days p.i. in which collected samples were PCR positive (top section), contained viable virus (middle section), and the maximum viral load found for each individual.

PCR Positive Range (days p.i.)								
	C-MPX 1	C-MPX 2	C-MPX 3	C-MPX 4	W-MPX 1	W-MPX 2	W-MPX 3	W-MPX 4†
Blood	3	12–18	-	-	12	-	12–15	12*
Nasal swab	3–27	3–42	3–42	6–18	3–42	12–21	3–56	6–12*
Oral swab	3–21	12–27	6–24	6–18	6–42	9–15	9–56	6–12*
Rectal swab	3–12	18	9–18	6–18	12–24	9–15	12–15	6–12*
Scarification site swab	3–27	3–56	6–35	3–24	3–56	3–49	3–49	3–12*
Secondary lesion swab	3–24	6–24	6–21	6	15–35	9–15	6–42	6
**Viable Virus Shedding Range (days p.i.)**								
Nasal swab	3–12	12–24	6–15	15–18	3–24	12–21	18–24	8–12*
Oral swab	3–15	9–24	6–18	9–15	6–18	9–15	9–18	6–12*
Rectal swab	3–9	-	9–15	12–15	12–24	9	12–15	9–12*
Scarification site swab	3–18	6–24	6–15	6–12	3–24	6–18	6–27	6–12*
Secondary lesion swab	3–12	6–15	12–21	-	15–18	-	15–18	-
**Maximum Viral Load (p.f.u./ ml)**								
Nasal swab	3.13X10^4^	4.55X10^4^	8.96X10^3^	2.46X10^2^	9.63X10^4^	3.66X10^2^	8.96X10^4^	3.73X10^4^
Oral swab	2.16X10^5^	6.42X10^5^	8.21X10^4^	2.16X10^4^	7.99X10^6^	7.09X10^5^	1.34X10^6^	3.81X10^7^
Rectal swab	3.21X10^4^	-	6.19X10^4^	4.48X10^1^	5.90X10^1^	5.67X10^2^	2.09X10^4^	3.21X10^2^
Scarification site swab	5.82X10^4^	1.64X10^7^	7.99X10^6^	6.94X10^5^	6.64X10^6^	3.51X10^7^	1.71X10^7^	1.05X10^8^
Secondary lesion swab	2.69X10^2^	3.88X10^4^	8.73X10^2^	-	5.97X10^1^	-	6.27X10^3^	-

* W-MPX 4 died on day 13 p.i., thus, no information is available for this animal after this day.

**Fig 3:**
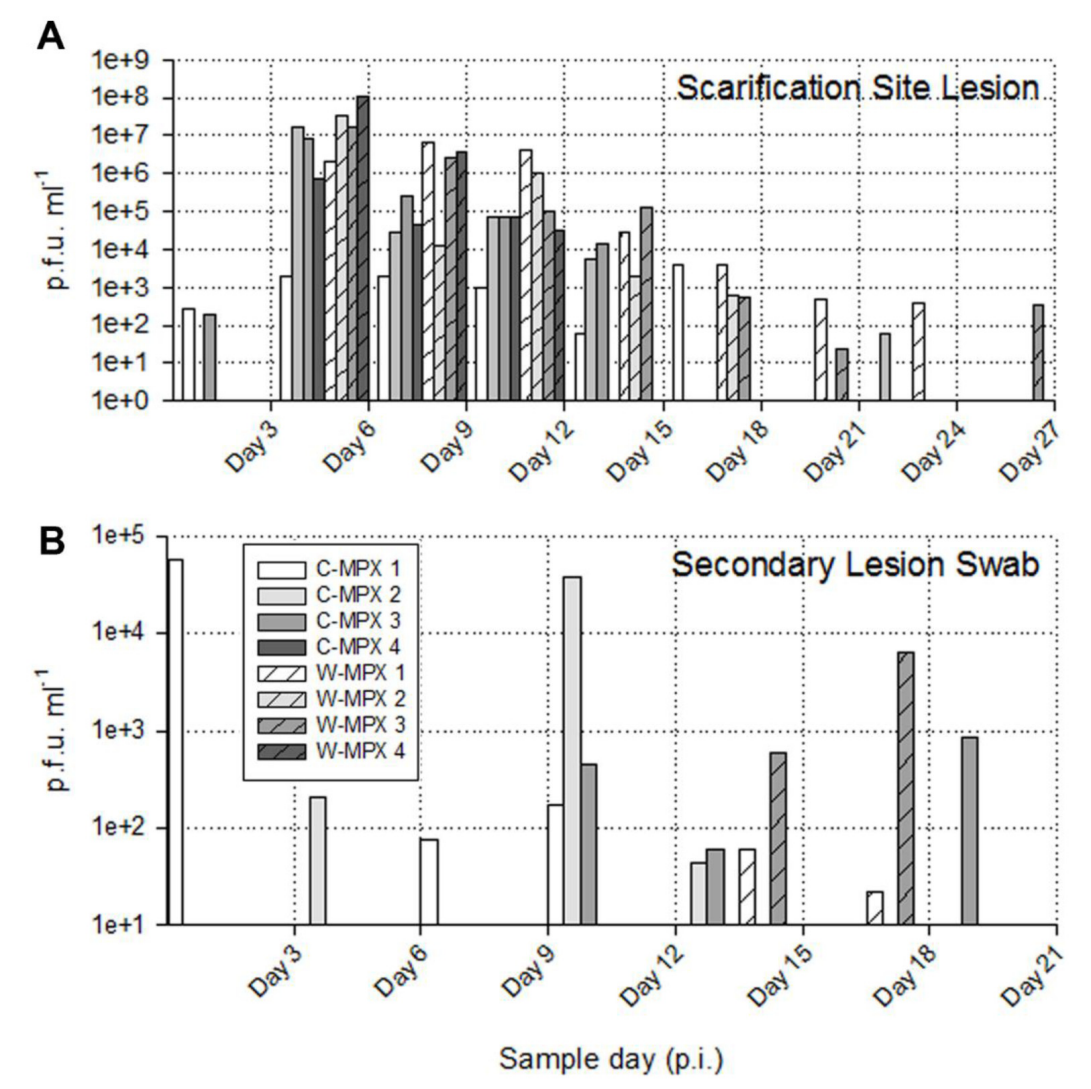
Viable viral load (pfu/ml) for each experimental animal found in scarification site (A) and secondary lesion swabs (B) throughout the study. (A) The maximum viral load from the scarification site was 1.05X10^8^ in an animal challenged with West African MPXV and 1.64X10^7^ for an animal challenged with Congo Basin MPXV. No viable virus was found in sample days after day 27 post infection (p.i.) (B) Lower loads of viable virus were recovered from secondary lesion swabs during a shorter period of time (day 3 p.i. to 21 p.i.); the maximum viral load was 3.88X10^4^ for an animal infected with Congo Basin MPXV and 6.27X10^3^ for an animal infected with West Africa MPXV.

**Fig 4:**
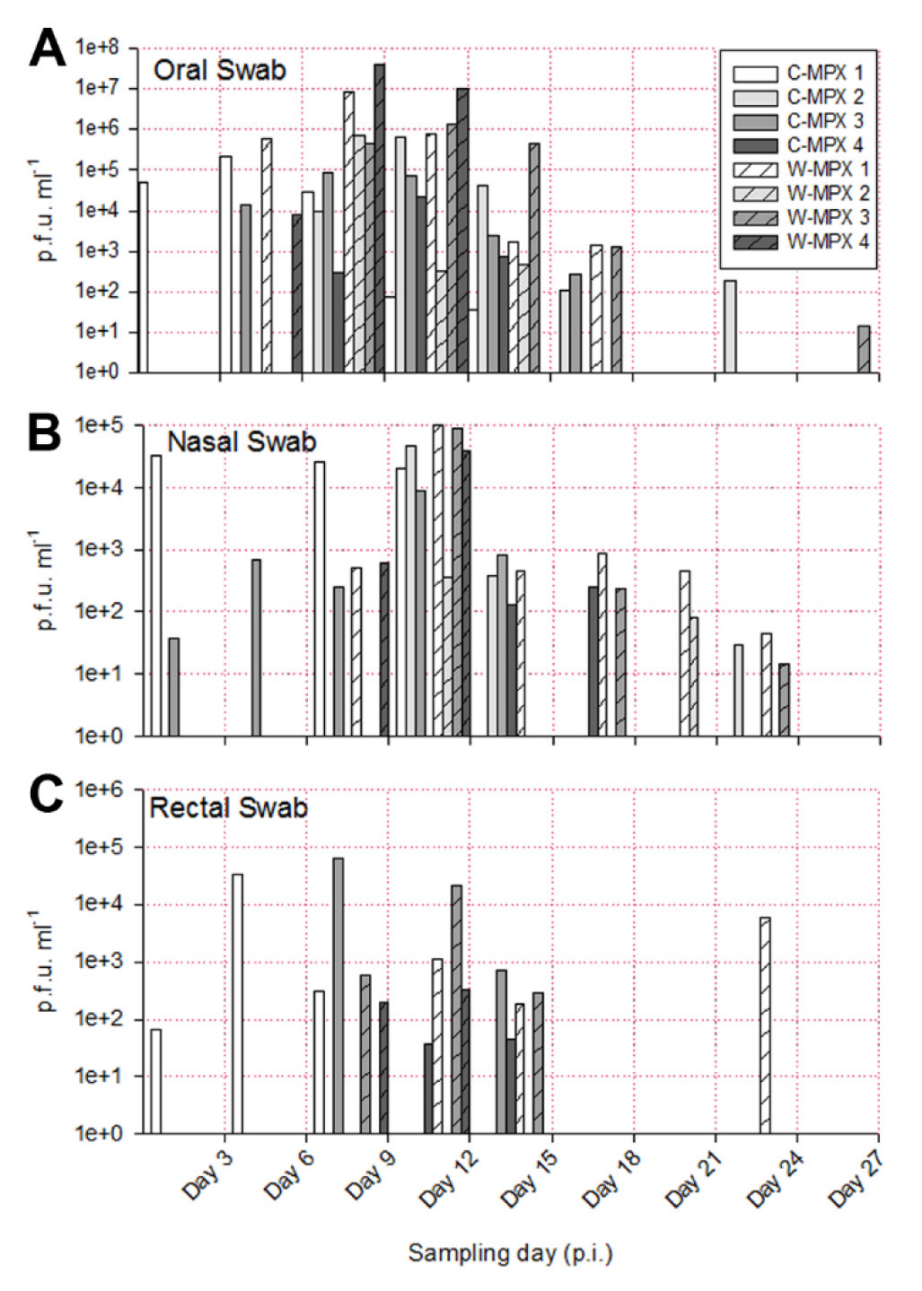
Viable viral load (p.f.u./ml) for each experimental animal found in oral (A), nasal (B) and rectal swabs (C) throughout the study. Higher viral loads were obtained from oral swabs than from rectal or nasal swabs. No viable virus was found in sample days after Day 27 post infection (p.i.).

The length of viral shedding was similar to viral DNA results, with some animals/samples having a delay in time between viral DNA positivity and viable virus detection. Infectious viral particles from at least one sample were first obtained from one C-MPX animals (C-MPX 1) and one W-MPX animals (W-MPX 1) on day 3 p.i. and from all experimental animals by day 6 p.i. (for at least one sample; Fig 3 and Fig 4). Viable virus could not be detected from one rectal swab that was positive for viral DNA (C-MPX 2). Additionally, viable virus could not be detected from secondary lesion swabs from three animals that were positive for viral DNA (C-MPX 4, W-MPX 2 and W-MPX 4). Cessation of viral shedding from all animals/samples had occurred by day 27 p.i. The mean number of shedding days was not significantly different between viral clades from the oral, nasal, lesions and rectal swabs and time of shedding in days for all samples can be seen in Table 3.

The shedding of viable virus was most prevalent in swabs of the inoculation site on day 6 p.i. (10^8^ pfu/ml) followed closely in magnitude by oral and nasal swabs 10^7^ and 10^5^ pfu/ml for C-MPX and W-MPX challenged animals, respectively; with peak levels seen on days 9 and 12 p.i. then decreasing steadily until the last detection on day 24 p.i. for C-MPX and day 27 p.i. for W-MPX (Figs 3 and 4). Loads of virus from rectal and scarification swabs are also depicted in Fig 3 and Fig 4.

**Fig 5:**
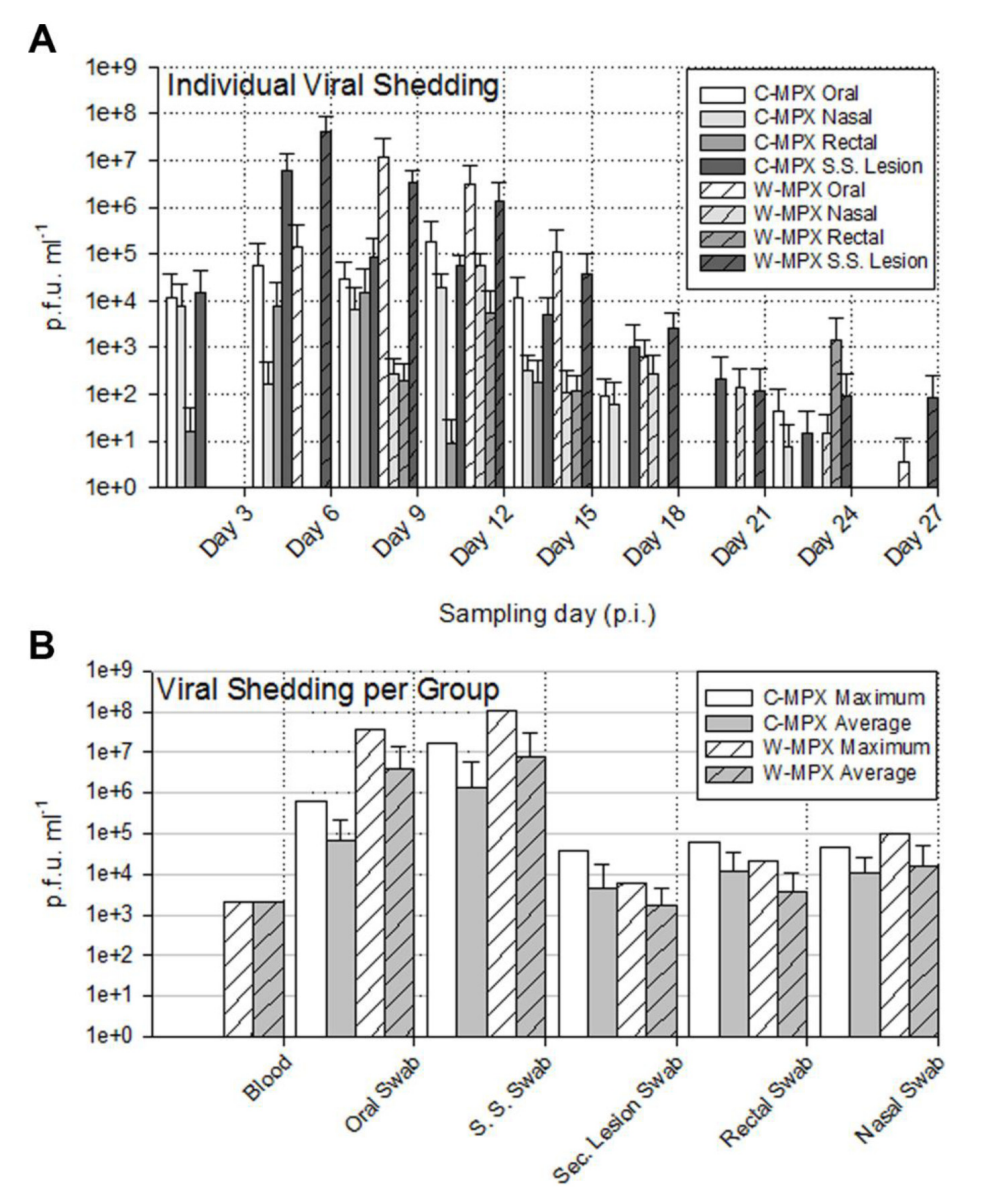
Average and maximum viral loads from MPXV challenged *Cricetomys*. (A) Average viral load per individual sample type and experimental group at each sampling day; no viable virus was found in sample days after Day 27 post infection (p.i.). (B) Maximum and average viral load for each sample type and experimental group throughout the entire study. Error bars represent one standard deviation.

Fig 5 summarizes the maximum and average viable virus obtained from each sample type by individual sample (Fig 5A) and by experimental group (Fig 5B). Statistical comparison, using the Wilcoxon rank sum test, of maximum viable virus found in blood samples (p = 0.4), oral (p = 1), scarification site (p = 0.8), secondary lesion (p = 0.66), rectal (p = 0.8) and nasal (p = 0.67) swabs between experimental groups showed no significant differences.

### Serology

**Fig 6:**
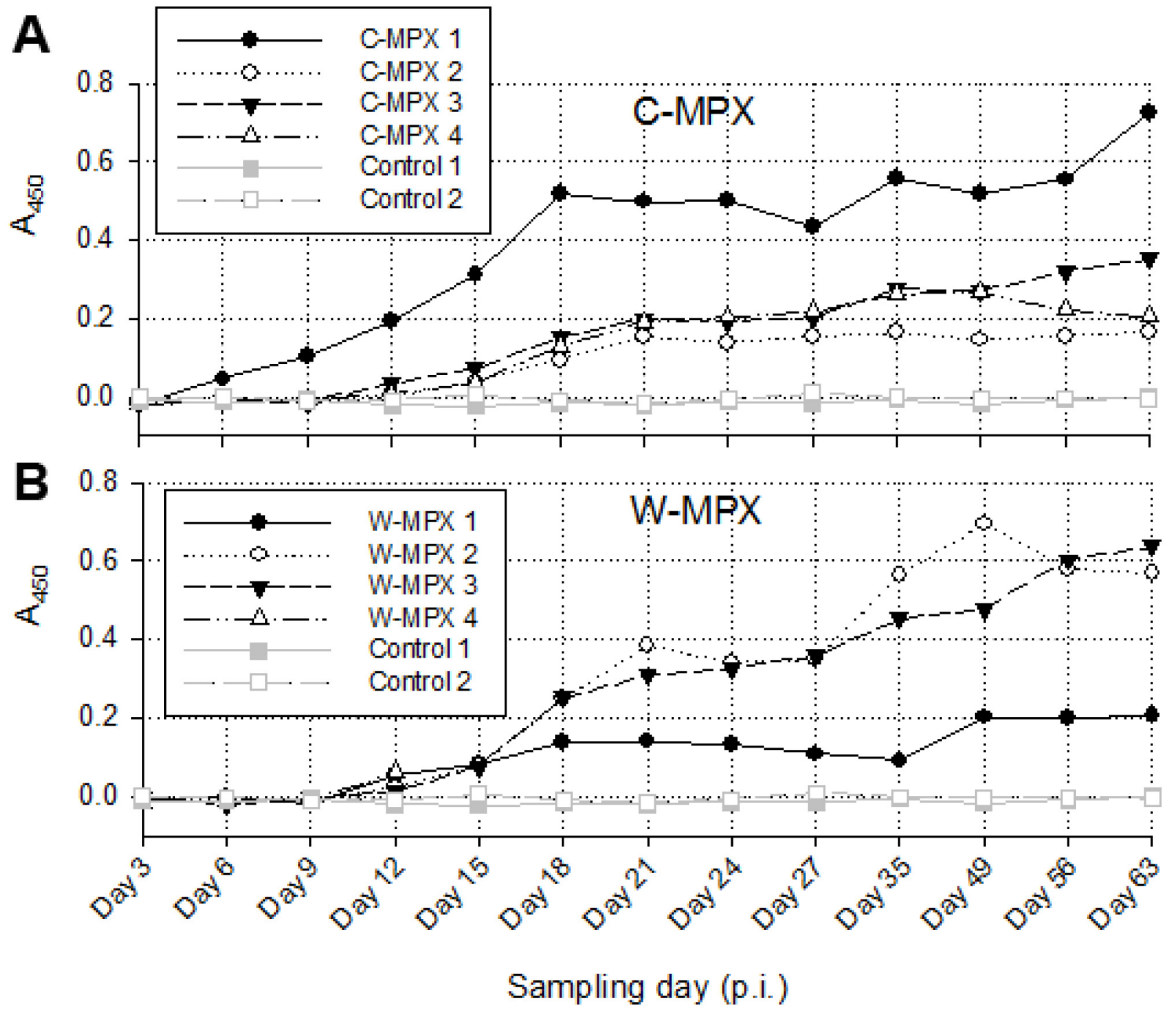
Immune response in *Cricetomys* after MPXV challenge. Absorbance values measured at 450nm from ELISA assays for both experimental groups: C-MPX (A) and W-MPX (B). No statistically significant difference was found between experimental groups (Wilcoxon signed test p-value = 0.3054). W-MPX 4 died on day 13 p.i., thus, no information is available for this animal after this day.

OPXV generic Ig antibodies were detected earliest in C-MPX 1 on day 6 p.i., (this was also the animal that first developed generalized cutaneous lesions on day 3 p.i.) (1). This individual also developed the highest level of antibody response of the C-MPX group with levels increasing most rapidly until day 18 p.i. and continuing to increase through day 63 p.i. (Fig 6). All other MPXV challenged animals developed a detectable antibody response by day 12 p.i.. Antibody levels varied between individual animals but differences were not significantly different between viral strains. Ig antibody levels fluctuated but increased abruptly for most animals until day 21 p.i.; after this initial increase there was a slower but continued increase in antibody titer or plateau at study end. The W-MPX animal that died on day 13 had Ig antibody levels similar to the other animals in its experimental group at time of death.

## Discussion

MPXV continues to be an important human health threat, causing sporadic outbreaks within Africa as well as the potential to spread outside its endemic range, as evidenced by the 2003 US outbreak. Identification of MPXV reservoir(s) is important so the public can be informed of associated risks with handling and consuming those species of animals and to improve infection control measures. Additionally the search to identify a small animal model that closely resembles human monkeypox disease progression continues in order to allow evaluation of vaccines and therapeutics against systemic Orthopoxvirus infection [38]. Up until the relatively recent development of the prairie dog and CAST/Eij MPXV models, there has been a paucity of small-animal models for the study of MPXV; specifically a small-animal model that mimics systemic disease in humans including the development of the characteristic cutaneous lesions. Due to the FDA’s animal rule, having numerous animal models that can be used to test efficacy of therapeutics and anti-virals against a MPXV challenge, especially at time of rash onset, would be beneficial.

Through our experimental infection of *Cricetomys*, we have shown that these animals may well serve as a natural reservoir of the virus and additionally could be utilized as a relevant animal model for the study of MPXV. With the exception of the prairie dog MPXV model, no other small animal model develops the cutaneous lesions that characterize human disease. Through the current study, we have shown that *Cricetomys* develop these skin lesions after MPXV infection and therefore may be utilized for the study of therapeutics at time of rash onset. The time until secondary lesions developed after animal inoculation was 9–12 days with the exception of one animal which developed lesions at 3 days p.i. The 9–12 day incubation before lesion onset is similar to the prairie dog MPXV model as well as the time-course believed to occur after a human is infected. Additionally, we were able to infect the animals with a plausible amount of virus to that which likely occurs in a natural transmission setting via scarification (to mimic a bite/scratch from an infected animal) and infected animals shed large amounts of virus from multiple secretions. During the 2003 US outbreak, people became infected with MPXV due to bites/scratches from infected prairie dogs [40,46]. Although we do not have the data from human exposures in Africa, we can hypothesize that a bite or scratch from an infected animal can lead to infection in people and in other animals. Additionally, it is widely accepted that MPXV is less transmissible via an aerosol route than smallpox in people as well as in an animal model of MPXV [47,48]; therefore a route other than intranasal is probably the most relevant when studying MPXV transmission in potential reservoirs. Animals may be infecting other animals when fighting with each other as occurs during transmission of other viruses such as Hanta virus [49], or perhaps when sharing the same food source and/or bedding and therefore oral excreta is shared. Follow-up studies with additional challenge routes would be worthwhile to explore differences in disease progression in MPXV infected *Cricetomys* due to infection route. However, the results from the current study provide evidence that *Cricetomys gambianus (*and by inference, probably the closely related species, *Cricetomys emini)* should be further considered as a likely MPXV reservoir species as well as potential animal model of monkeypox disease.

Both MPXV strains caused an overt rash illness in *Cricetomys*, although the morbidity and mortality were slightly lower than that reported in non-African rodents; particularly when comparing animals challenged with C-MPX [50]. Although observational data noted more pronounced lesions in the W-MPX animals, decreased activity and weight loss in both experimental groups suggest that infection with either clade of MPXV produces a systemic infection that affects the normal behavior of the animals (i.e., they became more stationary and consumed less food), but infected *Cricetomys* did not become moribund during the periods when they were shedding infectious virus of either MPXV strain. Slight differences were observed in body temperatures of MPXV infected *Cricetomys*, with C-MPX animals having a more marked febrile period and temperature difference compared to the W-MPX groups. This could suggest a more robust immune response in the C-MPX animals; however no significant differences were seen in anti-OPXV antibody levels between groups, and the course of illness was similar.

Because these were wild-caught, genetically heterogeneous animals, it is not surprising that some differences in disease presentation and mortality were observed. One animal challenged with C-MPX had an earlier disease time-course with both primary and secondary lesions evident by day 3 p.i. The antibody response was also expedited in this animal with antibody detection occurring 6 days earlier than all other animals. Viral DNA and viable virus were first detected earlier in all samples compared to the other *Cricetomys*; however the peak loads seen were similar. There was only one animal that succumbed to disease, an animal challenged with W-MPX. Although the disease presentation until the animal perished on day 13 was similar to other animals, necropsy results revealed extremely high loads of virus within all tissues tested (10^6^–10^9^ p.f.u./g). It is possible that the animal suffered from multi-organ failure and resulting death due to these high loads of virus. It is interesting that only an animal challenged with W-MPX succumbed to disease, as it is the less virulent clade within people as well as other animal models. We have previously genotyped the *Cricetomys* used within this study and shown that these animals originated from West Africa [Mauldin et al. manuscript in draft]. Generally viruses are believed to become attenuated within an animal host after circulation within that hosts’ geographic range [51,52], therefore we believe that this one animal’s death was most likely not reflective of the W-MPX virulence potential within *Cricetomys*, but specific to this individual animal.

The viral loads from infected *Cricetomys* are similar to that seen in MPXV infected non-African rodent species such as prairie dogs and ground squirrels in other laboratory challenge investigations, as well as during the 2003 MPX outbreak in the United States [11,23,31,53]. This level of virus should be more than sufficient to achieve transmission to other rodents or humans that might come into contact with the infected animals (especially through a bite) or their excretions. For example, the maximum amount of virus shed from the lesions, nose, and mouth of infected *Cricetomys* (1.05X10^8^, 9.63X10^4^ and 3.8X10^7^pfu/ml, respectively) exceeds the amount of virus used to initially challenge these animals (4X10^4^ pfu/ml). Interestingly, detection of viral DNA for most animals was not seen within blood samples until day 12 p.i. It is possible that our sample collection (i.e. amount of blood taken and days at which blood was collected) was not adequate for viral detection in the blood sample. Therefore we may have missed the primary/transient viremia that is believed to occur in humans and other animal models [54] [Hutson et al. 2015 Manuscript accepted BioMed Research International]. The shedding of virus detected in rectal swabs was variable but consistently lower and for a shorter period of time with respect to the levels and duration seen in other samples. Viable virus was recovered in lower titers (6.72X10^1^-6.19X10^4^ pfu/ml) from the rectal swabs primarily between days 9 and 15 p.i., but out to day 24 p.i. for one W-MPX individual. It is important to note that these were not fecal samples, but were swabs of the rectum; thus, although this suggests that virus could be found in the fecal pellets, it is not possible to determine how much infectious virus would actually be shed in such a manner. The detection of viral DNA unsurprisingly parallels the levels of viable virus detected in all of the samples. However viral DNA was detectable for a longer period of time and lasted until day 56 p.i. for both viral strains, compared to the last viable virus being detected at day 27 p.i.. These later viral DNA positive samples are presumably the result of sheared non-infectious viral fragments which persist beyond the infectious (viral shedding) stages of illness. This finding highlights the necessity of virus culture in field and laboratory studies examining the presence of enzootic and zoonotic diseases.

The results of our study are consistent with work done by collaborators who used bioluminescent imaging (BLI) to assess Congo Basin MPXV challenge (intradermal and intranasal inoculation) of *Cricetomys* [Falendysz et al.; submitted for Publication PLoS Negl Trop Dis]. Although the authors found a difference in clinical disease presentation depending on the route of inoculation, all animals shed high loads of virus, similar to the findings reported herein. Additionally BLI analysis allowed the investigators to demonstrate replication of MPXV in both healthy and sick animals. Thus it was also concluded by our collaborators that *Cricetomys* could be a potential source of MPXV infection for humans.

Members of the Genus *Cricetomys* including *C*. *gambianus* are found throughout Sub-Saharan Africa, including many areas that are outside of the known range of MPXV. Currently there is no known mammal species whose distribution perfectly overlaps with the distribution of MPXV, and the results presented herein along with those of other laboratory studies dealing with terrestrial rodents, suggest that the maintenance and transmission cycle of MPXV in Sub-Saharan Africa may involve multiple rodent species that can amplify and transmit the virus both within and between other mammalian species including humans. Based on past serosurveys conducted in Africa, *Cricetomys* have shown evidence of anti-OPXV antibodies and OPXV DNA; additionally, during the 2003 US Outbreak, *Cricetomys* was found to be infected with MPXV [16,20,23]. Although it is likely that there is a complex relationship between multiple rodent reservoirs in the transmission and maintenance of the virus within Africa, the data from our laboratory findings agree with the African serosurveys and suggest that *Cricetomys* should be considered as, at least, one potential MPXV reservoir host species that is involved in the maintenance and transmission of MPXV.

## Supporting Information

S1 Table P-values of the Wilcoxon rank-sum test performed to compare temperature, activity and weight loss of animals challenged with the West African clade of MPXV (W-MPXV), the Congo Basin clade of MPXV (C-MPXV) and the Control group at each of the sampling days. Weight loss values are not available for days 3, 56 and 70 p.i. because day 3 p.i. was used as the base value to calculate weight loss and animals were not weighed on days 56 and 70 p.i.
(DOCX)
