# Supplementary material for: Laboratory Investigations of African Pouched Rats (Cricetomys gambianus) as a Potential Reservoir Host Species for Monkeypox Virus
Source: PLoS Negl Trop Dis. 2015 Oct 30;9(10):e0004013. doi: 10.1371/journal.pntd.0004013 (PMC4627651; doi:10.1371/journal.pntd.0004013)
Supplement: S1 Table — Weight loss values are not available for days 3, 56 and 70 p.i. because day 3 p.i. was used as the base value to calculate weight loss and animals were not weighed on days 56 and 70 p.i. (DOCX) [file pntd.0004013.s001.docx]

S1 Table. P-values of the Wilcoxon rank-sum test performed to compare temperature, activity and weight loss of animals challenged with the West African clade of MPXV (W-MPXV), the Congo Basin clade of MPXV (C-MPXV) and the Control group at each of the sampling days. Weight loss values are not available for days 3, 56 and 70 p.i. because day 3 p.i. was used as the base value to calculate weight loss and animals were not weighed on days 56 and 70 p.i.

|  | **W-MPXV vs. Control** | | | **C-MPXV vs Control** | | | **W-MPXV vs. C-MPXV** | | |
| --- | --- | --- | --- | --- | --- | --- | --- | --- | --- |
|  | Temperature | Activity | Weight loss | Temperature | Activity | Weight loss | Temperature | Activity | Weight loss |
| Day 3 | 0.2667 | 0.5333 | NA | 0.8 | 0.5333 | NA | 0.6857 | 0.3429 | NA |
| Day 6 | 0.2667 | 0.5333 | 0.8 | 0.5333 | 0.5333 | 0.5333 | 1 | 0.5857 | 0.8857 |
| Day 9 | 0.1333 | 0.8 | 0.1333 | 0.5333 | 1 | 0.1333 | 1 | 0.8857 | 0.4857 |
| Day 12 | 0.1333 | 1 | 0.1333 | 1 | 0.2667 | 0.2 | 1 | 0.2 | 0.6286 |
| Day 15 | 0.1333 | 0.2667 | 0.2667 | 0.8 | 0.2 | 0.2 | 0.6286 | 0.4 | 1 |
| Day 18 | 0.1333 | 0.1333 | 0.1333 | 0.8 | 0.2 | 0.2 | 0.6286 | 1 | 0.4 |
| Day 21 | 0.1333 | 1 | 0.2667 | 0.8 | 0.8 | 0.2 | 0.8571 | 0.8574 | 0.6286 |
| Day 24 | 0.1333 | 0.8 | 0.2667 | 0.8 | 0.8 | 0.2 | 0.6286 | 0.6286 | 0.8571 |
| Day 27 | 0.1333 | 0.8 | 0.2667 | 0.8 | 0.8 | 0.2 | 0.8571 | 0.8571 | 0.6286 |
| Day 35 | 0.1333 | 0.1333 | 0.5333 | 0.8 | 0.2 | 0.2 | 0.6286 | 1 | 0.6286 |
| Day 42 | 0.1333 | 0.5333 | 0.5333 | 0.8 | 0.8 | 0.4 | 0.6286 | 1 | 0.6286 |
| Day 49 | 0.5333 | 0.8 | 0.5333 | 1 | 0.4 | 0.4 | 0.6286 | 0.8571 | 0.2286 |
| Day 56 | 0.06029 | 0.8 | -- | 0.8 | 0.4 | -- | 0.7213 | 0.8571 | -- |
| Day 70 | 0.1333 | 0.5333 | -- | 0.8 | 0.8 | -- | 0.8571 | 0.6286 | -- |
